# P284: Performance measures imputed bundle of infection control to reduce the incidence of tuberculosis hospital general labor 2005-2012

**DOI:** 10.1186/2047-2994-2-S1-P284

**Published:** 2013-06-20

**Authors:** FM Ramirez Wong, ZR Díaz Tavera

**Affiliations:** 1 Tropical medicine, Minsa,Universidad Cientifica del Sur, Lima, Peru; 2 Epidemiology, Universidad Nacional del Callao, Callao, Peru

## Introduction

We identify causes of nosocomial transmission of tuberculosis in health workers, inadequate: preventative maintenance, provision, purchase / allocation, needs boxes, distribution and use of N95 respirators (R-N95) misuse of natural ventilation, overcrowding . We implement surveillance of cases of occupational tuberculosis (TBCO) from the Hospital Infection Control Committee implemented and maintained joint control strategies(BUNDLE).

## Objectives

To determine the cost of the most successful strategy in reducing the TIA-TBCO. Dosimar H0: The application of strategies are equally expensive to reduce the rate of TBCO.H1: applying at least two different intervention strategies are lower in cost TBC occupational rate.

## Methods

Intervention study, longitudinal economically valued each health intervention with the TIA-monthly TBCO implemented: 2008 was applied around the BUNDLE, compare the cost medium TBCO with TIA-l! proof Kruskall - Wallis.

## Results

With a confidence limit of 95% of TIA TBCO decreased from 1.154 to 231 per 100,000, with p = 0.416 decreased TIA-TBCO were equal to the 5 strategies employed. Using all the TIA- BUNDLE TBCO decreased 6 times, 87%, 14.038 cases were avoided and saved $ 20 million, were avoided losing 768 years on disability for work.

## Conclusion

Use entire BUNDLE, with all it's always more effective. It is an effective preventive estartegia.

Save money. Lessens disease risks and controls. Increase the profitability of economically active lives. Improving health.

## Competing interests

None declared
